# Intraindividual Variability of Boldness Is Repeatable across Contexts in a Wild Lizard

**DOI:** 10.1371/journal.pone.0095179

**Published:** 2014-04-14

**Authors:** Laura Highcock, Alecia J. Carter

**Affiliations:** Department of Zoology, University of Cambridge, Cambridge, United Kingdom; Liverpool John Moores University, United Kingdom

## Abstract

Animals do not behave in exactly the same way when repeatedly tested in the same context or situation, even once systematic variation, such as habituation, has been controlled for. This unpredictability is called intraindividual variability (IIV) and has been little studied in animals. Here we investigated how IIV in boldness (estimated by flight initiation distances) changed across two seasons—the dry, non-breeding season and the wet, breeding season—in a wild population of the Namibian rock agama, *Agama planiceps*. We found significant differences in IIV both between individuals and seasons, and IIV was higher in the wet season, suggesting plasticity in IIV. Further, IIV was highly repeatable (*r* = 0.61) between seasons and we found strong negative correlations between consistent individual differences in flight initiation distances, i.e. their boldness, and individuals' IIVs. We suggest that to understand personality in animals, researchers should generate a personality ‘profile’ that includes not only the relative level of a trait (i.e. its personality), but also its plasticity and variability under natural conditions.

## Introduction

**Figure 1:**
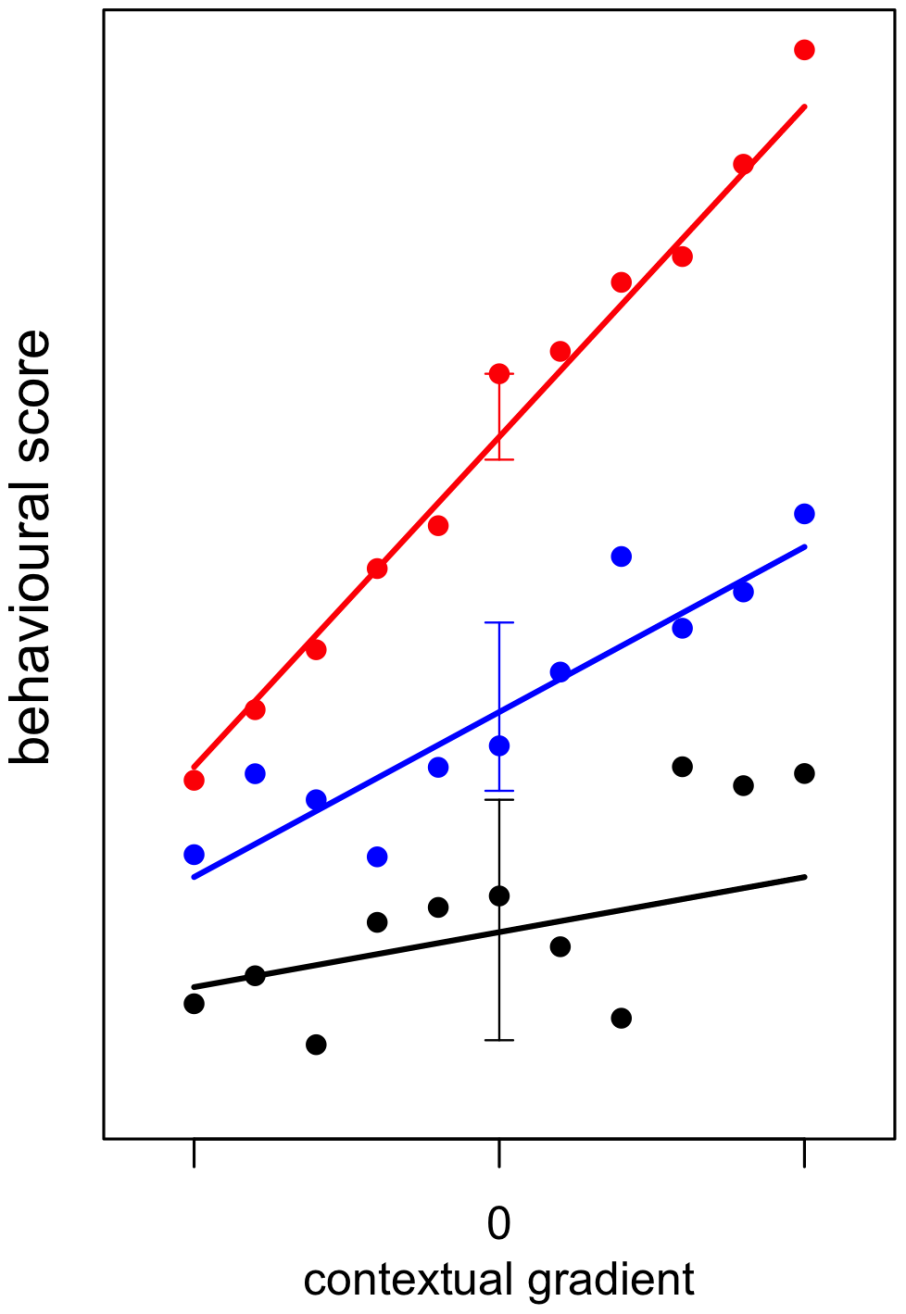
Three hypothetical individuals (black, blue and red) that have been measured 11 times over an environmental gradient, who differ in their personality, plasticity and intraindividual variability in behaviour. The points at which the lines cross 0 indicate the individuals' relative personalities; the slope of the line indicates the individuals' plasticities in the behaviour; and the arrows around the lines indicate the individuals' intraindividual variabilities (IIVs) in the behaviour. The black individual has a low value for its personality for this behavioural trait, low plasticity and high variability; the red individual has a high value for its personality, high plasticity and low variability; while the blue individual has average values for its personality, plasticity and variability for the behavioural trait.

Animal personality refers to low within- and high between-individual variation in behaviour and thus indicates behaviour that is repeatable through time [1]. This definition presents a challenge to ‘traditional’ views of behaviour not only because many behavioural traits are plastic—there is flexibility in behaviour in response to a change in context or situation—but also because of the tendency of individuals not to behave in exactly the same way when repeatedly tested or observed in the same context [2]. Some of this within-individual variability in behaviour is likely to be systematic changes due to, for example, familiarisation obtained during relatively short testing periods through processes such as habituation or sensory fatigue [2]. However, some within-individual variation remains once systematic variation has been accounted for, and these seemingly unpredictable fluctuations in behaviour have been defined as intraindividual variability (IIV) by psychologists [2], [3]. Thus, individuals may vary in: their relative position along a continuum for a behaviour (personality); how responsive they are to a change in context or situation (plasticity) and; how variable they are when measured repeatedly for a behaviour in one or more contexts or situations (IIV) (see Fig. 1 for a graphical representation of these ideas).

In humans, IIV in cognitive performance varies significantly with traits such as age [3], [4]—IIV is highest in very young individuals, reaches a nadir in early adulthood and increases in later adulthood [5]; is higher in people with certain brain disorders, such as dementia [5], [6]; and is lower in people with higher general cognitive performance [7]. In contrast to the psychological literature, there are few studies of IIV in animals and most of these have been conducted over a short time period and in a laboratory environment [2], [8]–[10]. Nevertheless, IIV has been demonstrated in behavioural traits such as boldness, exploration and activity [2], [8], [9], [11] and it is repeatable for activity in mosquitofish, *Gambusia holbrooki*, [9] and exploration in zebra finches, *Taeniopygia guttata* [8]. Further, there is a general trend that IIV of a behaviour is unrelated to the mean of a behaviour (Ward's damselfish *Pomacentrus wardii* and mosquitofish [2], [9]), or there is a weak relationship between IIV and the mean (hermit crabs *Pagurus bernhardus* [2]).

Few studies have included more than a single context or situation when studying IIV (context is a functional behaviour category and situation a set of conditions at a point in time [12]), but those that did have shown interesting trends. Hermit crabs that were exposed to a predator cue increased both the mean and the IIV of their latencies to emerge from their shell after a startle response and the author suggested that this was an adaptive response to dealing with higher perceived risk [10]. Further, female red-winged blackbirds, *Agelaius phoeniceus*, decreased their variability in provisioning behaviour as the provisioned chicks aged, and in response to where they had foraged for the chicks' provision [11]. Understanding whether IIV varies across contexts or situations in other species would allow important insights into whether IIV should be considered as a ‘trait’ (i.e. shows consistency through time and across contexts) that could be selected for, whether it fluctuates plastically similar to other behavioural traits, or whether it is independent of personality or plasticity. Growing evidence suggests that, as with plasticity, IIV is a personality trait in animals (i.e. is consistent through time, for example, see [9]), and based on studies in humans it is likely that there will be fitness implications of high or low IIV for different behaviours [13], [14]. This presents the possibility that individuals may vary in three aspects of their behavioural repertoires: their personality, plasticity and variability in behavioural traits. However, despite this possibility, IIV remains poorly studied in behavioural ecology.

Additionally, from a statistical point of view, the existence of significant differences between individuals in behavioural variability violates the assumption of equal variance which underlies many statistical models, questioning the reliability of many statistical tests commonly used in the study of animal behaviour [2] and highlighting the need to investigate how general this trend may be. Further, while studies of IIV in a laboratory may be better able to control variation in external stimuli, studies under natural conditions allow ecologically relevant inferences to be drawn from finding consistency or plasticity in IIV. If multiple measurements are taken [15] and observations randomised, reliable estimates of IIV could be made in the wild, and IIV's influence on other behavioural traits could be assessed. However, there is an important caveat to all studies of IIV, and especially those conducted on wild or free-ranging animals: an individual's estimate of IIV will include not only its variability in the behaviour, but also the error associated with measuring the behaviour. This error may include (unmeasured) variables that may affect behaviour [2], such as differences in: labile states, such as satiation; recent social experience, such as the presence of a conspecific; and the surrounding environment, such as visibility or food availability. It is thus possible that differences in the estimates of IIV may not measure intrinsic behavioural variability as anticipated, but are the result of ‘extrinsic’ heterogeneity. With this caveat in mind, we aimed to describe the relationship between personality and IIV across contexts in a wild, free ranging lizard, the Namibian rock agama, *Agama planiceps*.

The Namibian rock agama is an ideal study organism for investigating intraindividual variation in a wild population across a contextual or situational gradient. The onset of the rainy season presents a rapid change in both context and situation, as it simultaneously transitions from non-breeding to the breeding season (context) and from low to high resources (situation). While context and situation are conflated, this represents the natural ecology of the study organism [16]. Further, the agamas' boldness can be quantified rapidly and repeatedly in the wild without the need for trapping by measuring flight initiation distance (FID; the distance at which an individual flees from an approaching threat). A lower FID indicates an individual who is willing to allow the close approach of a potential predator and is thus indicative of greater boldness [17]–[19]. FID has been shown to differ between individuals and to be consistent through time and across seasons in the Namibian rock agama [16], [17]. However, while boldness is consistent across seasons in this species, its correlation with other behaviours as a component of a behavioural syndrome is not: In the wet-dry transition, a behavioural syndrome exists linking boldness (average FID) to the time the agamas spend ‘conspicuous’ (basking and moving) and its related benefits to food acquisition and territory size, while in the dry-wet transition, this syndrome no longer exists [16], [17]. Understanding how the IIV of FID in Namibian rock agamas fluctuates across seasons presents an interesting test of whether IIV may be linked to the personality trait boldness (if it correlates with boldness and is consistent through time) or rather whether it is plastic (if IIV changes across seasons in a similar fashion to the other behaviours that are linked to the putative behavioural syndrome). Using this unique system, we aimed to answer the following four questions: (Question 1, Q1) Do Namibian rock agamas show inter-individual differences in IIV; (Q2) given that agamas differ in their IIV of FID, is IIV consistent or plastic across seasons; (Q3) does boldness correlate with IIV, and (Q4) does IIV correlate with a proxy of fitness, specifically body mass?

## Methods

### Ethics statement

This research adhered to the Guidelines for the Use of Animal Behaviour for Research and Teaching (*Anim. Behav.* 2003. **65**, 249–255). This research was approved by the Australian National University Animal Experimentation Ethics Committee (permit number: F.ES.03.10) with permission from the Ministry of Environment and Tourism (Namibia) (permit number: 1529/2010).

### Study area and species

Data were collected from October to December 2010 in one population of Namibian rock agamas at Hobatere campsite in north-west Namibia (70°53037.74″S, 19°28031.35″E). The transition from the dry season to the wet is sudden; no rain (0 mm) was recorded until late in the day on the 15^th^ November. One to four mm of rain was recorded over the following 4 days and intermittently for the remainder of the study after the initial rains. Males increased their rates of signalling during territorial defence and courting to male and female conspecifics, respectively, from before to after the onset of the rains [16]. Therefore measurements preceding November 15 2010 were deemed ‘dry’ season measurements and after this date as ‘wet’ season [16]. Adult agamas are sexually dimorphic in colouration, and males were individually identified by natural variation in their colour patterns and other distinguishing features such as scars [17].

The boldness of individual male agamas was repeatedly assessed by measuring flight initiation distances. A single observer (AJC) approached each male on foot at a constant speed (4 km/h; measured using a GPS unit [eTrex, Garmin, Olathe, KS, USA]) after a 10 min observation period (performed as part of another study: [16]) from a distance of approx. 20 m (range 10–35 m) depending on the position of the agama in relation to the observer at the end of the observation period. Males were approached to test their FID when an observation period ended with the male basking prominently within his home range [17], or had been watched for at least 3 min in the case of those individuals (*n* = 2) that did not form part of the observational study (for details, see [16], [20]). The distance from the observer when the male fled was measured to the nearest 5 cm using a measuring tape. Smaller FIDs are indicative of higher boldness [17].

We identified and measured the FID of 47 individuals, 34 of which were tested more than 5 times and which form the basis of these analyses. Of these 34 individuals, 33 and 32 were present and measured at least 3 times in the dry and wet seasons, respectively, and which form the basis of the analyses of between-season data. Finally, 31 of the males were common to both seasons.

### Trapping of agamas

Fifteen agamas were successfully trapped using a clap trap (45×45 cm). Clap traps (or clap nets) consist of two sides of netted mesh that close together when sprung, trapping the individual between the two sides of netting. The clap trap was baited with insect larvae, and the trap was sprung either by the researcher using a string attached to the release mechanism, else the trap was automatically sprung when the agama bit at large larvae in the release mechanism. The clap trap was positioned on the ground or a similar flat surface at the base of a rock or ledge that the target agama was occupying. If the agama moved away from the trap, the trap was repositioned closer to the agama. Males' masses (to the nearest g, measured with a 50 g spring scale) and male snout vent lengths (SVL; to the nearest mm, measured with digital callipers) were recorded. Males were released immediately after measurements were taken and no male was handled for longer than 180 s.

### Statistical analysis

Our analyses took four approaches in line with our four questions. First, we estimated individuals' variances in FID using the following modelling approach. We used the natural log (ln) transformation of FID to satisfy assumptions of normality as the response variable. Ambient temperature and time of day do not affect FID in this species for this dataset [20]; thus we did not include these variables as fixed effects in the following models. However, FID is known to decrease in this species as habituation occurs [16], [20], thus to control for systematic changes in behaviour over time, we included observation number as a fixed effect in these analyses. Further, we have previously assessed whether individuals habituated at different rates between seasons (by including a random slope for individual in interaction with season), and whether being trapped affected subsequent FIDs, but found no evidence for either of these [16], [20]; thus we include observation number as the only fixed effect in the ln-FID models used to estimate IIV in the current study. We fitted Markov chain Monte Carlo linear mixed models (MCMCglmms) with the package ‘MCMCglmm’ [21] in the R environment [22] with 170 000 iterations, a burn in of 70 000 and thinning of 10 iterations with an inverse Wishart prior. Standard MCMCglmm diagnostic checks [23] were performed for all models. Individual identity was included as a random effect. To estimate individuals' variances in FID, we fitted MCMCglmms with heterogeneous residual variances by setting the residual covariance matrix to estimate a variance for each level of the individual intercept. We used the posterior modes of the variance estimates from this model as the estimates of IIV.

We tested whether there were inter-individual differences in IIV (question 1, Q1) by determining whether allowing the random effect of individual identity to have heterogeneous variances explained a significant amount of variation in the model. To do this, we compared the difference in the deviance information criteria (ΔDIC) from models with homogeneous and heterogeneous variances for individual identity. A ΔDIC >10 would indicate that heterogeneous variances for identity explained a substantial amount of variation in the ln-FIDs, and thus there was evidence for intra-individual variability in FIDs [24]–[26]. However, as the R structure specified in the prior for heterogeneous variances model differed from that specified in the homogeneous model, these results should be treated with caution [23].

Second, we considered differences in IIV between seasons. To assess whether there was a population-level change in IIV across seasons (Q2), we calculated the predicted individual variance estimates from MCMCglmms (see above) using the data from the dry season and data from the wet season separately. As we have previously found that male agamas spend more time exposed to predators in the rainy season [16] and IIV is known to increase in response to perceived predation risk [10], we tested whether there was an increase in IIV from the dry to the rainy season using a one-tailed, paired t-test for the 31 individuals common to both seasons. We then calculated the repeatability, *r*, of IIV between seasons using the package rptR [27] with the ‘MCMC’ method to investigate whether IIV was consistent across seasons (Q2). A confidence interval that does not come close to 0 would indicate a repeatable behaviour.

Third, we considered whether IIV was personality dependent. As a measure of boldness, we used the posterior mode estimates of the individual intercept as the measure of each individual's ‘average’ FID. IIV was estimated as the posterior mode of the variances as outlined above and was ln transformed to satisfy assumptions of normality. We used a linear model to investigate whether IIV correlated with FID overall, and separately in each season (Q3).

Finally, we investigated whether IIV correlated with a proxy of an individual's fitness, specifically their mass (Q4). To do this, we compared the masses of individuals to their overall IIVs using a linear model.

## Results

**Figure 2:**
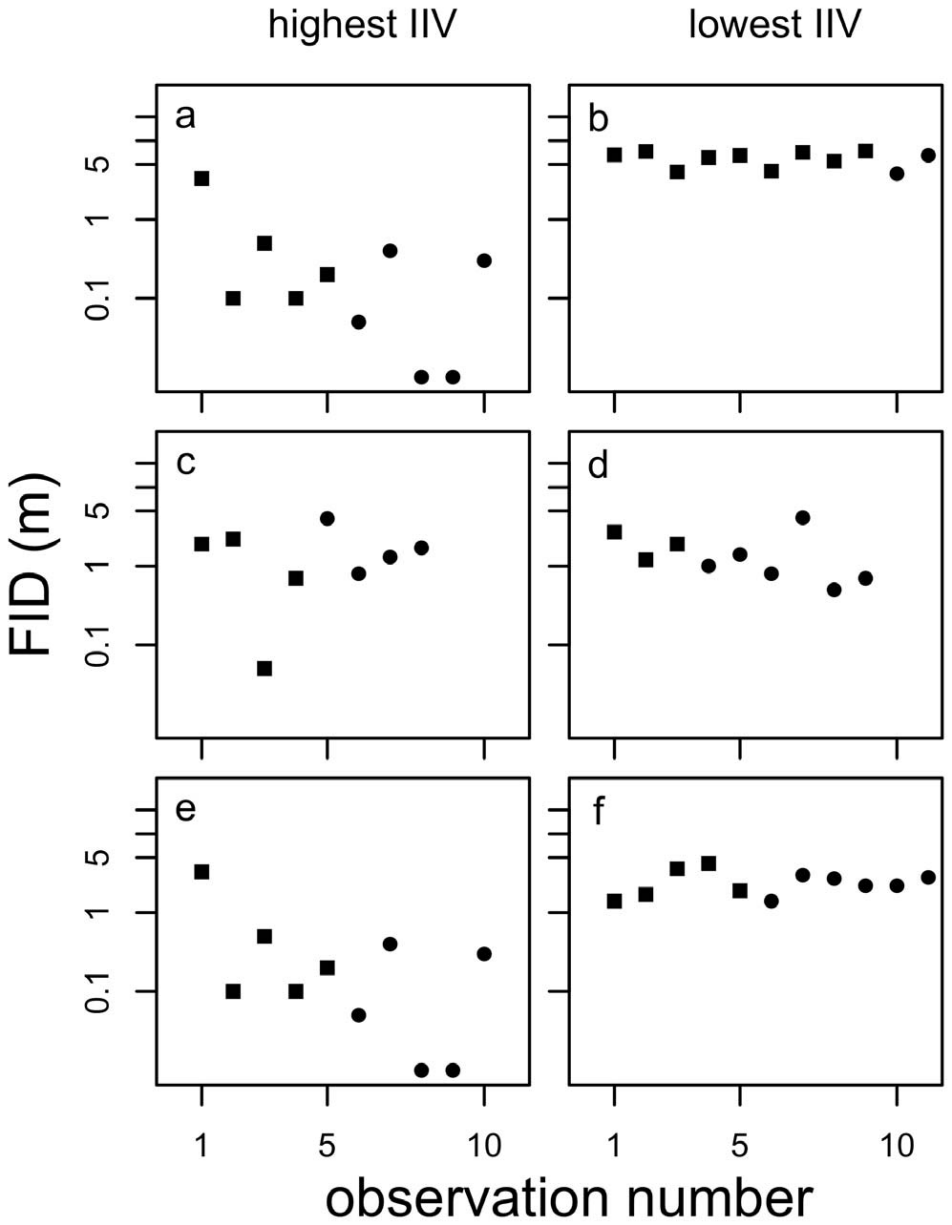
Flight initiation distance (FID) (m) over successive observations for the most (highest IIV; a, c and e) and least (lowest IIV, b, d and f) variable individuals overall (a, b) and in the dry (c, d) and wet (e, f) seasons. Note that the most variable individual overall was also the most variable individual in the wet season. Measurements taken during the dry and wet seasons are indicated by filled squares and circles, respectively.

We first investigated whether individuals varied in their IIV by comparing models with homogeneous and heterogeneous variances for the random intercept for individual (Q1, Fig. 2). In all cases, the models with heterogeneous variances were better supported than the models with homogeneous variances. Over both seasons, there was a 39.4-fold difference in IIV among individuals (Q1: ΔDIC = 114.8, *n_ind_* = 34, *n_obs_* = 383). There was greater variability among individuals within seasons: In the dry season, there was a 77.7-fold difference in IIV (Q1: ΔDIC = 98.2, *n_ind_* = 33, *n_obs_* = 203) whilst after the transition to the wet season there was a similar 77.4-fold difference in IIV (Q1: ΔDIC = 41.0, *n_ind_* = 32, *n_obs_* = 178).

**Figure 3:**
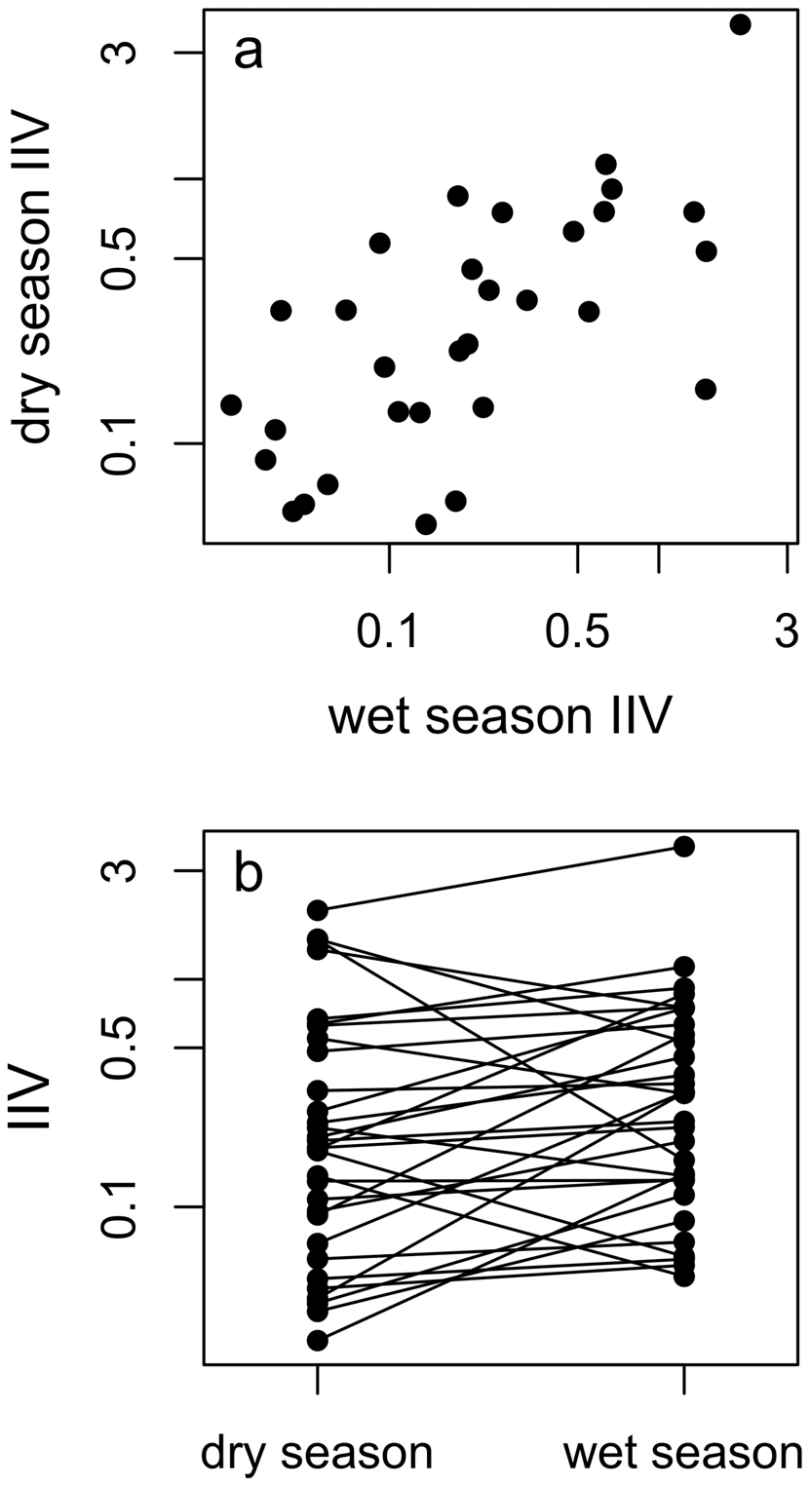
The relationship between intraindividual variability (IIV) estimated in the dry and wet seasons. (a) shows a scatterplot of the relationship while (b) shows the individual responses across seasons.

Next, we investigated whether IIV was consistent or plastic across seasons for the 31 agamas that were common to both seasons. As predicted, the population-level IIV was significantly higher in the wet season compared to the dry season, though this difference was relatively small (Q2, Fig. 3, difference  = 0.32, *t* = −1.90, CI = >0.04, df = 30, *p* = 0.03). While this suggests that IIV changed between seasons, this was not supported by a further two-tailed t-test (CI = −0.67–0.02, df = 30, *p* = 0.07), and, although in the predicted direction, this finding should be treated with caution. However, IIV was highly repeatable between seasons (*r* = 0.61±0.12, CI = 0.33–0.78) (Q2, Fig. 3).

**Figure 4:**
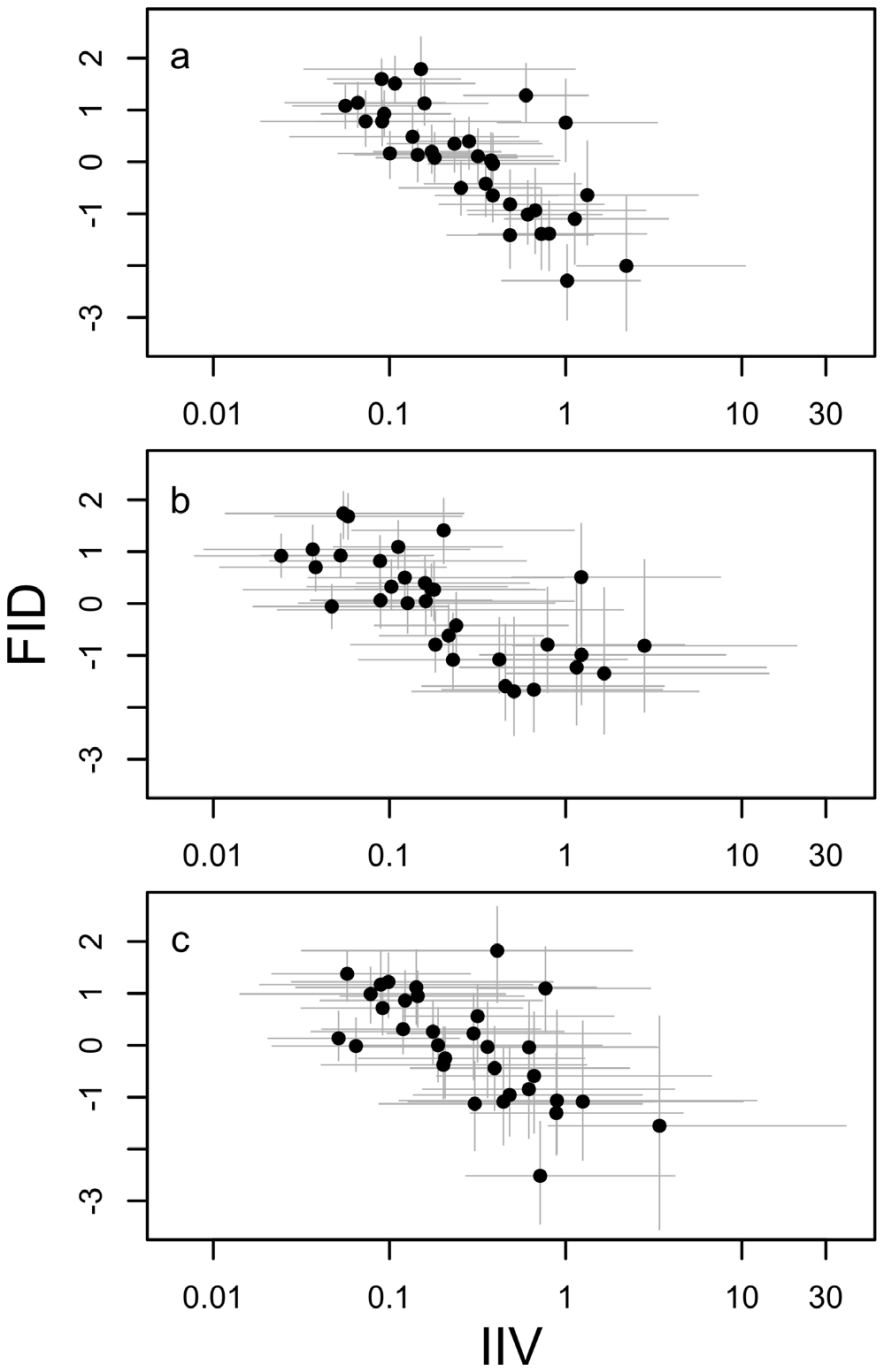
The relationship between the individual estimates of flight initiation distance (FID) and intraindividual variability (IIV) in FID for (a) the entire study period, (b) the dry season and (c) the wet season. Indicated by grey lines are the 95% highest probability density estimates for the individual FIDs and IIVs.

Third, we tested whether IIV depended on boldness i.e. the average individual FIDs which were estimated as the posterior modes of FID (Q3). There were strong correlations between the posterior modes for FID and IIV for individuals that remained despite using ln-FID to quantify IIV (Fig. 4). Overall, higher mean FIDs correlated with lower IIV estimates (β±s.e. = −0.30±0.06, *t* = −5.22, *p*<0.001; Fig. 4a). The same trend was evident on the subset of data from the dry season (β±s.e. = −0.27±0.08, *t* = −3.50, *p* = 0.001; Fig. 4b) and in the wet season (β±s.e. = −0.32±0.11, *t* = −2.94, *p* = 0.006; Fig. 4c). This suggests that bolder individuals have higher intraindividual variability in FID compared to shyer individuals.

Finally, however, there was no correlation between overall IIV and a proxy of fitness, the mass of the agamas (Q4: linear model: β±s.e. = 2.21±2.13, *t* = 1.04, *p* = 0.32).

## Discussion

We asked several questions about the nature and consequences of the IIV of boldness in wild Namibian rock agamas across two seasons. We found evidence that intraindividual variability in flight initiation distances differed significantly among individuals both within seasons and overall. Additionally, though IIV increased slightly between seasons, IIV was highly repeatable, suggesting that IIV could be considered a ‘trait’ in this species. Further, we found strong correlations between IIV and boldness. Overall and in both the dry and the wet seasons, shyer individuals—those with higher mean FIDs—were less variable—had lower IIVs. However, there was no effect of IIV on the mass of the agama. Below we discuss IIV in the existing framework of personality and plasticity and its implications for this framework before considering our findings in more detail.

We found significant, repeatable differences in IIV across individuals, suggesting that the FID of an individual cannot be summarised accurately by a single average value. To our knowledge, this is the first study to demonstrate the repeatability of IIV in the wild and that it can be plastic, and only the second study to show repeatability of IIV [9]. Our results support previous suggestions that, instead of using a single measure, the behaviour of an individual should be quantified by a distribution of values, specifically the relative level (personality), plasticity and variability of the behaviour [2], [9]. This approach raises an intriguing problem about ranking individuals on a continuum of shy-bold based on FID (or any other measure of a personality trait). That is, it is unclear whether an individual that has consistently moderate-to-low FID should be classed as bolder than a highly variable individual that predominately has high average behaviour but occasionally has extremely low FID, exhibiting extreme boldness [28].

IIV was slightly lower in the dry season than in the wet season. We have previously suggested that there are different selection pressures acting on these agamas which resulted in behavioural plasticity between the wet and dry seasons [16]. Specifically, we highlighted the importance of defending mates and territory in the wet, breeding season compared with predator avoidance in the dry, non-breeding season [16]. Indeed, males spend more time exposed to predators in the wet, breeding season [16] and we predicted the agamas would increase their IIV because of this. This finding is in line with a study on hermit crabs in which individual increased their IIV for their latencies to emerge from their shell in response to higher perceived predation risk [10]. Further, the cost of having a high FID—responding earlier to a predator—will be higher in the breeding season due to lost fitness enhancing opportunities such as courting females and defending a territory [29]. Individuals would thus be expected to have lower FIDs in the wet season, which was found to be the case [16]. We could further predict that individuals would be more responsive in the breeding season, adjusting their FIDs to the prevailing social conditions [29], [30], resulting in higher IIV in the wet, breeding season. We found support for this hypothesis here; however future work should determine whether this finding is consistent across species or in the same species across years. As we mentioned in the Introduction, an important caveat to our findings is that the agamas were observed solely in one environment, their own territories, and these changed little between the two seasons (although we note here that some individuals substantially shifted their home ranges between seasons). It is possible that IIV was determined by the agamas' habitat and not by an ‘intrinsic’ behavioural variability specific to an agama. Future research could investigate whether IIV is consistent across alternative gradients (rather than just season), which would suggest that IIV is a characteristic of an individual, rather than a plastic response to a situation common to all individuals. Future experimental manipulations could further test this hypothesis by measuring individuals' IIVs in different social and environmental conditions: if IIV was consistent in, for example, multiple different territories, there would be a strong argument for an ‘intrinsic’ IIV.

There was a strong correlation between IIV and the boldness of an individual. Bolder individuals (those with lower FID) were always more variable than shy individuals [2]. This was unlikely an artefact of differing habituation speeds between individuals of differing boldness as the agamas habituated at the same rate [16]. This finding is in contrast to two other studies on IIV: in mosquitofish there was no relationship between average levels of activity and IIV of activity [9] and in hermit crabs there was a positive relationship between the mean latency to emerge and the IIV of emergence times [2]. However, individuals with lower FIDs may be at higher risk of predation if they allow a predator to approach nearer and may benefit from having more unpredictable escape behaviour [31], in this case, showing higher IIV in flight initiation distances. This hypothesis is unsupported by the findings in hermit crabs mentioned above [2], however, and requires further investigation.

As highlighted by Biro and Adriaenssens [9], no evolutionary theory has been developed for understanding or predicting individual differences in IIV. While our results add to a small but growing literature describing IIV in animals, they also highlight some avenues of theory which may be of potential use to the field. For example, protean escape behaviour is predicted to decrease predation risk [31], and, as described above, IIV increases with increased perceived predation risk [10], which we also suggest may be responsible for the slight increase in IIV in the wet season in this study. The IIV literature could thus take advantage of the established literature on predator-prey interactions [32]–[34], especially that of prey vigilance behaviour in which there are well-known relationships between vigilance and perceived predation risk [35], well-developed theory predicting these relationships [36], and a developing appreciation of ‘unpredictability’ in vigilance behaviour [37] that could be used to make predictions regarding the plasticity and direction of change in the IIV of antipredator behaviours. Alternatively, a more general approach could consider individual differences in state. A recent model predicted responsive (variable) decision-making should occur as a function of an individual's state, for example, their size, and the reliability of information about the environment [38]. This model may thus provide testable predictions about when to expect differences in intraindividual variation in behaviour between individuals (with respect to individual differences in state) and within individuals (with respect to temporal changes in the reliability of information).
